# Applying machine learning to explore the association between biological stress and near misses in emergency medicine residents

**DOI:** 10.1371/journal.pone.0264957

**Published:** 2022-03-08

**Authors:** Sonnet Xu, Judith E. Arnetz, Bengt B. Arnetz

**Affiliations:** 1 Troy High School, Troy, Michigan, United States of America; 2 Department of Family Medicine, College of Human Medicine, Michigan State University, Grand Rapids, Michigan, United States of America; Universiti Malaysia Pahang, MALAYSIA

## Abstract

Physician stress is associated with near misses and adverse medical events. However, little is known about physiological mechanisms linking stress to such events. We explored the utility of machine learning to determine whether the catabolic stress hormone cortisol and the anabolic, anti-stress hormone dehydroepiandrosterone sulfate (DHEA-S), as well as the cortisol to DHEA-S ratio relate to near misses in emergency medicine residents during active duty in a trauma 1 emergency department. Compared to statistical models better suited for inference, machine learning models allow for prediction in situations that have not yet occurred, and thus better suited for clinical applications. This exploratory study used multiple machine learning models to determine possible relationships between biomarkers and near misses. Of the various models tested, support vector machine with radial bias function kernels and support vector machine with linear kernels performed the best, with training accuracies of 85% and 79% respectively. When evaluated on a test dataset, both models had prediction accuracies of around 80%. The pre-shift cortisol to DHEA-S ratio was shown to be the most important predictor in interpretable models tested. Results suggest that interventions that help emergency room physicians relax before they begin their shift could reduce risk of errors and improve patient and physician outcomes. This pilot demonstrates promising results regarding using machine learning to better understand the stress biology of near misses. Future studies should use larger groups and relate these variables to information in electronic medical records, such as objective and patient-reported quality measures.

## Introduction

Stress adversely impacts physician performance with potentially adverse effects on the quality of care that the patient receives. Chronic stress and stress-related disorders are associated with impaired work function and decreased work engagement, such as sick leave and presentism [1]. Many studies have examined the prevalence of burnout among physicians [2], to sometimes alarming results. Almost 1 in 2 US physicians have symptoms of burnout, which has been associated with increased odds of being named in a medical malpractice suit [3]. For emergency room physicians, job-induced stress can be particularly high with implications for cognitive fatigue and clinical practice [4].

Several studies have associated physician stress to adverse medical events and medical errors [2, 3]. However, there are limited studies on possible neurophysiological mechanisms that would explain a causal link between stress and near misses and related medical events. Studies have shown that stress may influence executive function [5] and may therefore be a major contributor to adverse medical decision making. Grueling work schedules and cognitively demanding work likely also contribute [6].

### Near misses

Although adverse medical events are relatively rare, and therefore difficult to study, near misses are more common [7]. According to the National Academy of Medicine, reporting and addressing adverse events and near misses is critical to enhance patient safety [7]. However, few studies have examined the relationship between objective measures of physiological stress and near misses [8]. A near miss has been defined as “any process variation that did not reach the patient, employee or visitor, but for which a recurrence carries a significant chance of a serious adverse event”. Because they are more common, near misses serve as a reliable marker for overall safety [7].

### Machine learning

Machine learning is a branch of artificial intelligence and lies at the intersection of statistics and computer science. It utilizes a variety of statistical, probabilistic, and optimization methods to allow computers to “learn” from past examples and accumulate experience. Briefly, statistics focuses on learning from data and computer science focuses on developing efficient computer algorithms [9]. While statistical models have a focus on inference, machine learning models work more towards prediction of future outcomes in situations that have not yet been observed [10]. Machine learning has proven to have many potential applications in the biomedical field [11] and is part of a growing trend towards more personalized medical treatment.

One of the most notable uses of machine learning has been in the field of cancer research, where it has been applied for over 30 years [12]. Among well designed studies, machine learning has shown to substantially improve the accuracy (15–25%) in predicting cancer susceptibility, recurrence, and mortality [13]. A recent meta-analysis comparing theory-driven and machine learning prediction of suicide showed that machine learning could provide superior performance in the prediction of suicide ideation, attempts, and death in comparison to the suboptimal performance across all surveyed theoretically driven models [14]. Similarly, promising results have been found in predicting stress in medical professionals using machine learning models, which increased the accuracy of stress prediction up to 20.8% as compared to traditional statistical procedures [15].

Particularly within the field of Emergency Medicine, machine learning has also been applied to the detection of physician stress. Using wearable sensors that collected biometric data using accelerometry, electrodermal activity, etc., an algorithm was designed to identify physician self-reported periods of stress and demonstrated a potentially promising method to facilitate biologically based stress research [16]. Machine learning has also shown promise in studies of cognitive decline, with machine learning derived biomarkers being validated as a potential surrogate biomarker for intervention studies [17]. These promising results seen across a variety of disciplines, including those directly relevant to this study, indicate that machine learning methods have strong potential to be applied as a tool to enhance the ability to predict near misses.

### Biomarkers of stress

In order to better understand the physiological basis of near misses, the current study focused on the catabolic stress hormone cortisol and the anabolic, anti-stress hormone dehydroepiandrosterone sulfate (DHEA-S). Cortisol has been explored more extensively as a biomarker for stress. However, in this study of near misses, we complemented modeling of the individual stress hormone cortisol with the anti-stress and neuroprotective hormone, DHEA-S, as well as the cortisol to DHEA-S ratio [18]. Both hormones are central to the biological stress response and DHEA-S is believed to attenuate the adverse effects from cortisol on brain neurons. Cortisol is a biomarker for neurodegenerative disease versus DHEA-S, which is neurorestorative, protecting brain cells from toxic cortisol levels [19]. Thus, the absolute concentration of these hormones as well as the ratio between the two offers a more complete measure of the body’s stress pattern.

The objective of this study is to use a machine learning approach to establish a function to predict near misses using the ratio between the catabolic stress hormone cortisol and the anabolic, anti-stress hormone dehydroepiandrosterone-sulfate, DHEA-S, along with the absolute blood concentrations of both. Compared to statistical methods, machine learning was a better approach for identifying and describing various patterns in our study, as the potentially noisy data and variable clinical settings could otherwise interfere in traditional statistical models. Results of this pilot study applying machine learning to explore the physiological basis of near misses could provide a foundation for further studies of mechanisms involved in proactively linking physiological stress to adverse clinical decision-making processes.

## Methods

**Fig 1:**
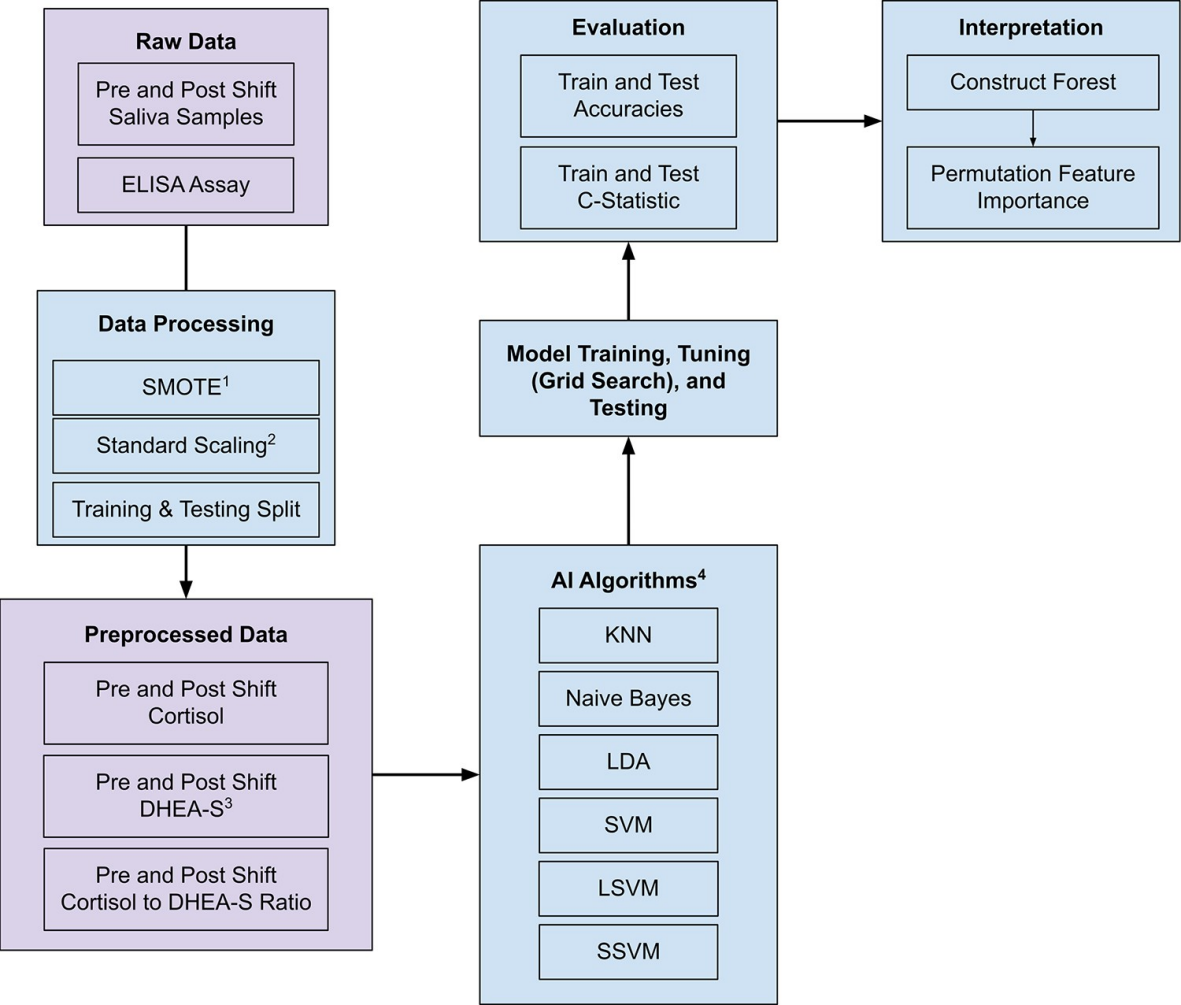
Study methodology. ^1^Synthetic Minority Oversampling Technique. ^2^Standard Scaling removes the mean and scales to unit variance. ^3^Dehydroepiandrosterone-sulfate. ^4^The AI Algorithms mentioned are the k-nearest neighbors, Naïve Bayes, linear discriminant analysis, support vector machine (with the default radial bias function kernels), support vector machine with linear kernels, and support vector machine with sigmoidal kernels respectively.

This study was a secondary analysis of de-identified data collected in 2015–2016 for a study of self-reported and biological stress and near misses among 28 emergency residents [7]. The original study was approved by the Wayne State University Institutional Review Board (Protocol # 1204010830) and the Detroit Medical Center (DMC) Research Office. Data collected contains information on demographics, years of medical training, post-stress recovery strategies, perceived skills, complexity mix of patients seen during the shift, and resident-reported near misses. Study participants were residents in emergency medicine working at the DMC, Michigan, USA, a level 1 trauma center. Only a portion of the originally collected data was used in this analysis and is provided in S1 File. In particular, the biomarker data used (described below) were the only features that the model was trained on. Fig 1 depicts an overview of the methods employed.

As a brief overview, after receiving raw saliva samples and performing the ELISA assay, the data was then processed with Synthetic Minority Oversampling Technique (SMOTE), scaled, and split into training and test dataset. The preprocessed data was then fed into a variety of AI algorithms which were tuned by means of grid searching for optimal hyperparameter values, and iteratively testing. These finalized models were then evaluated. Finally, the results of the models were interpreted with permutation feature importance. Each step is further detailed below.

### Biomarker data

Saliva samples were drawn for 28 physicians immediately before the start of a shift and after the end of a shift. Approximately 1 mL of saliva was collected by passive drooling and placed on ice, later being transported to the lab for biomarker analysis with commercially available enzyme-linked immunosorbent assay (ELISA) kits. See [7] for more comprehensive methods description.

The machine learning models exclusively used the biomarker data collected, i.e., pre-shift cortisol concentration, pre-shift DHEA-S concentration, pre-shift Cortisol DHEA-S ratio, post-shift cortisol concentration, post-shift DHEA-S concentration, and post-shift Cortisol to DHEA-S ratio. The biomarker data aforementioned were the six initial features that were used to train the model. Table 1 depicts descriptive information about all data used for the model.

**Table 1:** Biomarker information (n = 28).

Biomarkers	Median Value	Standard Deviation
Cortisol Pre-shift [μg/dL]	4501.00	3023.48
Cortisol Post-shift [μg/dL]	1499.00	1200.10
DHEA-S Pre-shift [μg/dL]	317.00	488.13
DHEA-S Post-shift [μg/dL]	236.00	217.19
Cortisol DHEA-S Ratio Pre-Shift	14.50	13.40
Cortisol DHEA-S Ratio Post-Shift	7.63	9.52

To prepare the dataset for the machine learning models, it was first oversampled with Synthetic Minority Oversampling Technique (SMOTE) to balance and increase the frequency of near miss datapoints within the dataset. It was then randomly split into training and testing datasets, with the training dataset comprised of 70% of the data, and the testing dataset comprising 30% of the data. Datapoints with any missing data were then dropped. All data was then normalized by removing the mean and scaling to unit variance.

### Model implementation

Several machine learning models were used and compared to explore the possible relationship between pre and post shift concentrations of cortisol, DHEA, and the cortisol to DHEA ratio and the outcome variable resident-reported near misses. Tested models included linear discriminant analysis (LDA); K nearest neighbor (KNN); Naïve Bayes (NB); and support vector machine (SVM) with linear, radial bias function (RBF), and sigmoid kernels. For detailed information regarding these models, please refer to [20].

The linear discriminant analysis model was implemented using singular value decomposition solver, to deal with the issue of small sample size problem with data of high dimensionality. The KNN algorithm was tuned at different values of k, and the final implementation had a k value of 5. The distance metric used for the KNN model was the Minkowski metric, which is a generalization of the Euclidean distance and Manhattan distance. This metric was employed with a power parameter of two, which is equivalent to the standard Euclidean metric. For the support vector machine with a polynomial kernel, we used a polynomial of the third degree.

To tune the various hyperparameters of the estimators employed, we searched the hyper-parameter space for the best cross validation score. For each model, we defined a grid of possible parameter values for tunable hyperparameters (such as k value for KNN, etc.). All possible combinations of parameter values were explored, and the best combination was obtained. This exhaustive grid search aided in the determination of the final models.

The models and all the accompanying data preparation methods were all constructed and performed with Python, version 3.7 (Python Software Foundation Inc.). All models were assessed for accuracy and c-statistic. Following this, model interpretation was done by means of feature importance ranking, which was assessed with a separately constructed forest of trees.

Permutation feature importance was the metric used when determining feature importance and is defined to be the decrease in a model’s accuracy score when a single feature is randomly shuffled. By breaking the connection between a feature and the prediction target, permutation feature importance allows the assessment of how much a model depends on a feature. With a model *m* and a dataset *D*, a reference accuracy of *s* is first computed. Then, for each feature *j* within *D*, *j* is randomly shuffled to generate a distorted version of *D*. For each repetition k in *1*, *…*, K: the *s*_*k*,*j*_ of model *m* on the distorted *D* is then recomputed. The feature importance is then defined as:

$$i_{j}=s-\frac{1}{K}\sum_{k=1}^{K}s_{k,j}$$

As all employed features were demonstrated to positively improve the accuracy of the models, all features were retained following the feature importance stage. Permutation feature importance was used primarily to further understand the relative importance of all the features to the model, and how to different biomarker measures compared to each other in terms of accuracy contribution.

### Model assessment metrics

The machine learning algorithms were compared and assessed using the accuracy and c-statistic. Accuracy refers to the number of instances that the model predicted correctly out of all instances evaluated on. It is calculated with the following equation.


$$Accuracy=\frac{Number\;of\;Correct\;Predictions}{Total\;Number\;of\;Predictions}$$

A true positive (TP) is when a model correctly predicts a positive class. In the case of this study, the model correctly predicted that the physician reported a near miss. A true negative (TN) is when the model correctly predicts the negative class, in our case, when a physician did not report a near miss. False negatives (FN) are when the positive class has been misclassified, i.e., a physician with a reported near miss classified as one without a near miss. False positives (FP) are when the negative class has been misclassified, i.e., when a physician that did not report any near misses has been classified as one that has.

A receiver operating characteristic (ROC) curve shows the relationship between the true positive rate and false positive rate at all classification thresholds. The true positive rate (TPR) is defined as

$$TPR=\frac{TP}{TP+FN}$$

The false positive rate is defined as

$$FPR=\frac{FP}{FP+TN}$$

The ROC curve shows tradeoff between specificity and sensitivity in a binary classifier, plotting the true positive rate (TPR) against the false positive rate (FPR) at various thresholds. The area under the ROC curve, abbreviated AUC, measures the degree of separability and is a metric that evaluates how capable a model is of distinguishing between the different two classes. AUC can range from 0 to 1. An AUC of 0.0 means the model has 100% false predictions, while an AUC of 1 means the model correctly predicts every case. AUC is also referred to as c-statistic in some literature, which is how it is referred to for the rest of this paper. AUC is a common indicator of model performance and has been used frequently in many machine learning studies [15].

## Results

Of the 28 physicians in the study sample, the majority were male (71.4%). One quarter (25.0%) reported having experienced near misses during their shift. After oversampling, the dataset consisted of 48 datapoints, 24 of each class, that is, those that reported near misses during the shift and those that did not. The results of fitting all the models on the dataset are shown in Table 2.

**Table 2:** Accuracies of different models tested.

Model	Training Accuracy	Test Accuracy	Training C-Statistic	Test C-statistic
SVM	85%	80%	86%	75%
LSVM	79%	80%	80%	75%
SSVM	73%	73%	74%	72%
LDA	79%	73%	78%	73%
KNN	76%	73%	77%	69%
NB	79%	73%	79%	72%

SVM is the default support vector machine (with radial bias function kernels). LSVM is the support vector machine with linear kernels and SSVM is the support vector machine with sigmoidal kernels. LDA represents the linear discriminant analysis model, KNN is the k-nearest neighbors model, and NB is the naïve bayes model.

**Fig 2:**
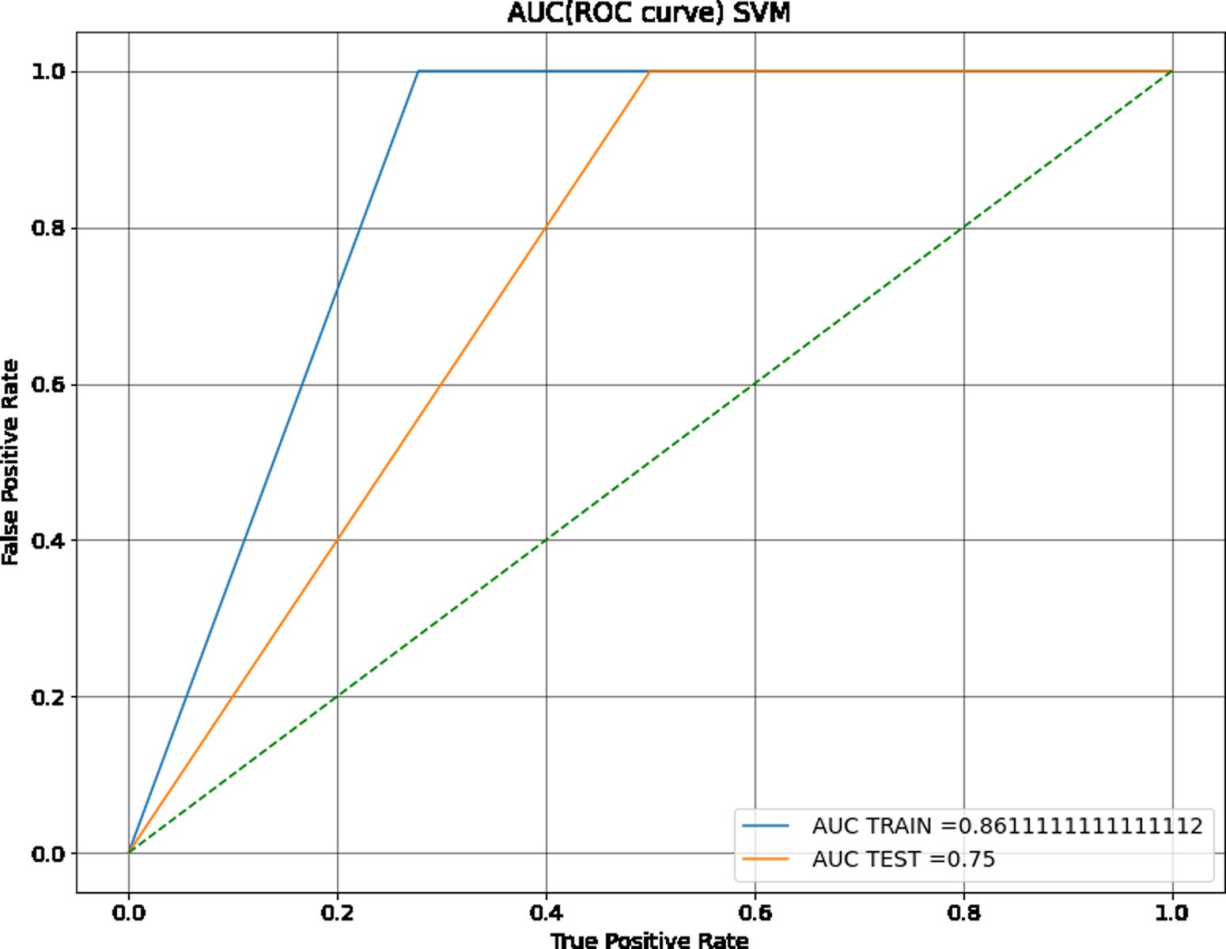
ROC curve of SVM with RBF kernels. This SVM model performed similarly across training and testing datasets.

**Fig 3:**
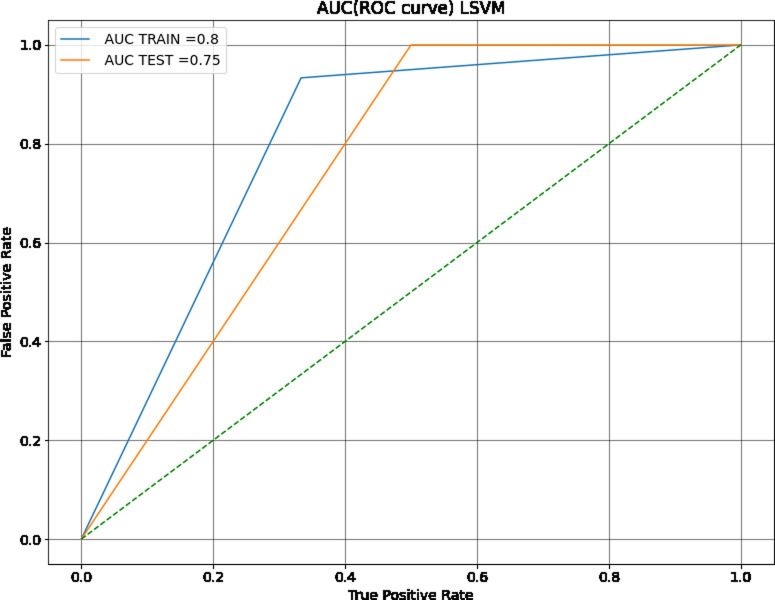
ROC curve of SVM with linear kernels. The SVM with Linear Kernels showed a smaller difference between training and testing datasets than the SVM with RBF kernels.

Most of the models had comparable accuracies. SVM with RBF kernels (the default, abbreviated as SVM from here forward), SVM with linear kernels (LSVM), and LDA models performed the best, with training accuracies of 89%, 79%, and 87% respectively. The SVM also had the highest training c-statistic, 86%, and the receiving operating characteristic (ROC) curve for this model is shown in Fig 2. The SVM with linear kernels or linear SVM (LSVM) had a training c-statistic of 80% and a test c-statistic of 75% (Fig 3). With this metric, the LSVM also outperformed all other models except the SVM. Performance, in this case, was assessed by comparing metric values and determining which one was higher. Of the various support vector machine kernels tested, the SVM with sigmoidal kernels (SSVM) had the poorest performance when compared to the SVM and LSVM by measure of accuracy and c-statistic.

**Fig 4:**
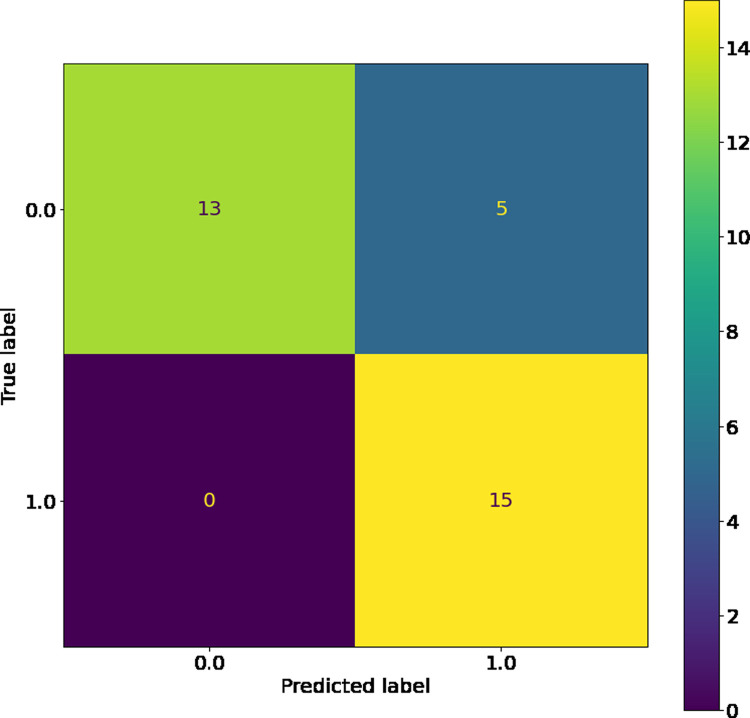
Confusion matrix of SVM with RBF kernels on training data.

**Fig 5:**
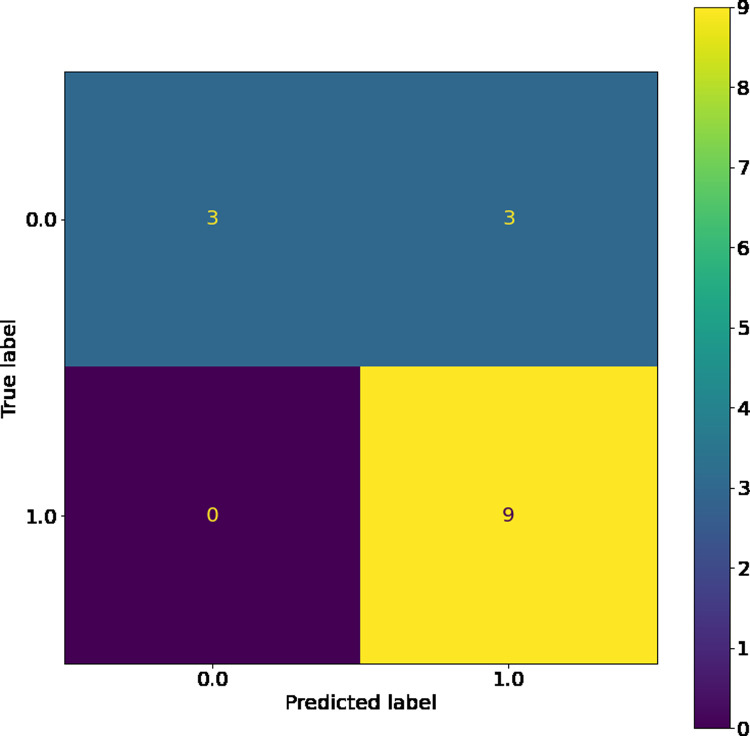
Confusion matrix of SVM with RBF kernels on testing data.

**Fig 6:**
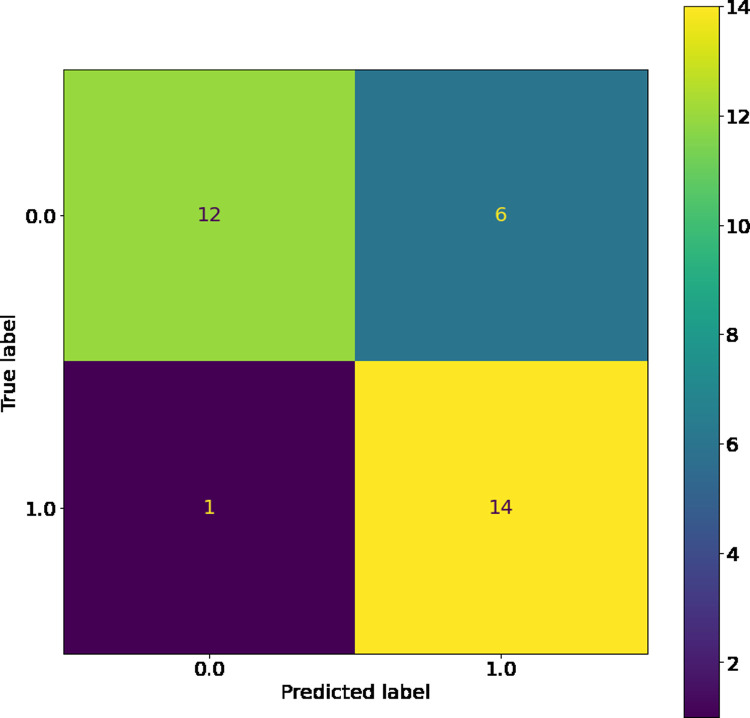
Confusion matrix of SVM with linear kernels on training data.

**Fig 7:**
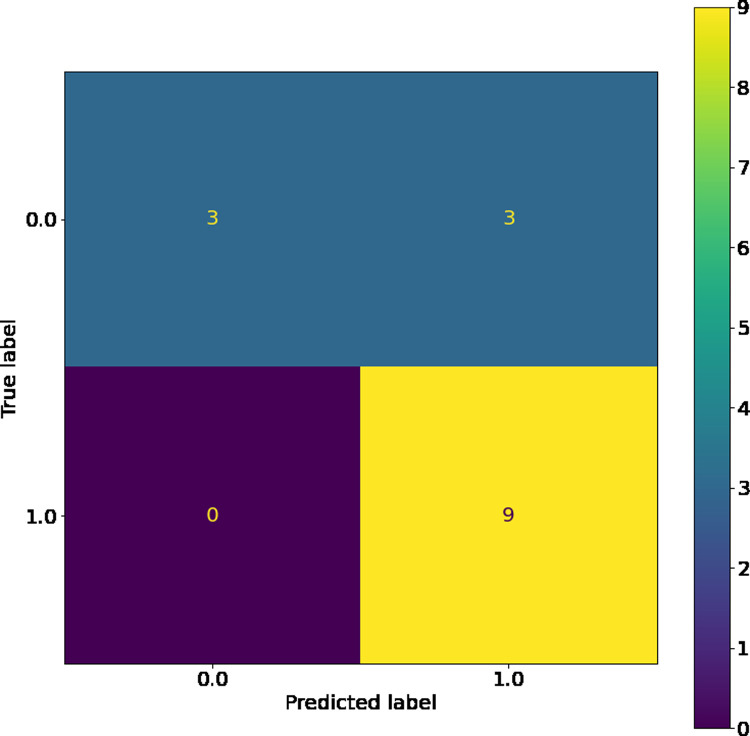
Confusion matrix of SVM with linear kernels on testing data.

The SVM, while having the highest training accuracy, showed a larger difference between training and test dataset c-statistic than other comparable models, so it was therefore not chosen as the best model. However, as seen in its confusion matrices (Figs 4 and 5), the SVM has good resistance against false negatives, and did not have a single instance of them in the training or testing dataset. The LSVM was selected as the best model, as it had high performance that was stable across the training and testing datasets. The confusion matrix for this model on the training and testing data sets can be seen in Figs 6 and 7. All in all, the SVM and LSVM had similarly good performance, with respect to multiple metrics.

**Fig 8:**
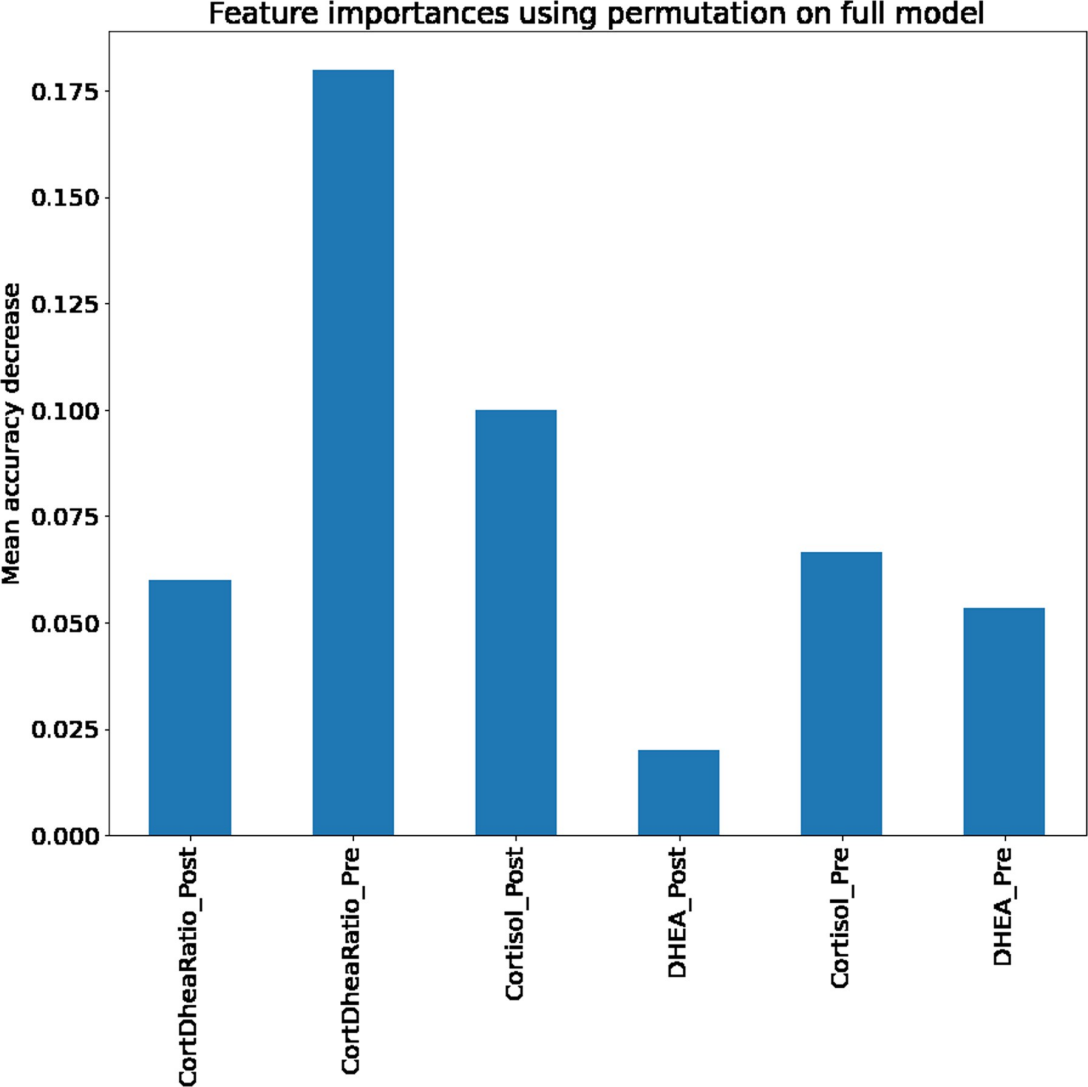
Permutation feature importance. Using a forest model we were able to determine the relative importance of each feature used in the models.

The results of permutation feature importance rankings from a separately constructed tree model are shown in Fig 8. Using five iterations, we found the cortisol to DHEA-S ratio measured before the shift to be the most important predictor of near misses, and it was ranked significantly higher than the post shift cortisol to DHEA-S ratio. Consistent with this observation, the DHEA blood concentration was a more predictive factor than the post shift DHEA blood concentration. However, we interestingly found that the post shift cortisol concentrations was more predictive than its pre shift counterpart.

## Discussion

The objective of this investigation was to use machine learning to explore the relationship between relative and absolute concentrations of stress and anti-stress biomarkers to predict near misses and evaluate the performance of these models. Our study experimented extensively with SVM, and the SVM with linear kernels and with RBF kernels were among the top performing models in this study. The LSVM did not indicate overfitting and was selected as the best model. Feature importance methods showed that the pre-shift cortisol to DHEA-S ratio was the most significant predictor of near misses.

As SVM are robust in the presence of noise, they predict well in this application, which explains their overall high performance. The LDA model also performed comparably, and since SVM are extensions of LDA models, this likely explains why the models performed relatively similarly. No model significantly lagged in prediction accuracy. NB seems like a clinically promising model for further investigation as it has good performance, interpretability, and efficiency. Relative blood concentrations of the biomarkers, or the cortisol to DHEA ratio, were shown to be significant to predict stress related near misses. As discussed in the introduction, the ability of machine learning models to perform beyond the level of inference and generalize to future conditions allow for effective clinical implementation.

The inclusion of the pre and post shift cortisol and DHEA-S blood concentrations, as well as the pre and post shift cortisol to DHEA-S ratio may seem redundant, as the machine learning models should be able to infer this relationship and determine if it is correlated with near misses. However, the explicit addition of both the pre and post shift cortisol increased the accuracy of all models tested. This likely reflects a strong relationship between frequency of near misses and the cortisol to DHEA ratio. The pre-shift cortisol to DHEA ratio was chosen as the most important predictive feature by using a separate tree to measure permutation feature importance, and this ratio was shown to be positively correlated with near misses. Contrastingly, post shift cortisol and post shift DHEA-S were more predictive than their pre shift counterparts.

LDA is a robust classification method that was tested for this study because of its predictive ability and simplicity [21], as it does not require extensive tuning of hyperparameters. It has also been shown to produce good classification results for low dimensional data, the case for this study, and is useful in real world applications because of its fast prediction speeds and small memory requirements [22]. It requires relatively fewer samples; thus, it can be used with small sample sizes [23] and was therefore suitable for this study. LDA performed comparatively well, having a training accuracy of 79% and a validation accuracy of 73%. Particularly with the c-statistic of the LSVM, the training c-statistic, 80%, and the testing c-statistic, 75%, were quite comparable, which suggests that the model likely did not overfit, a concern when dealing with limited data.

Something of note is that while it had poorer accuracy, the LSVM seemed to have less of an issue with overfitting, as with both metrics (accuracy and c-statistic) the gap between the training and test dataset performance is more significant in the SVM with RBF kernels. KNN and NB models consistently performed similarly, with comparable accuracies and c-statistics for both training and testing trials.

The SVM is an extension of the LDA classifier and uses separating hyperplanes to distinguish between different classes of data and is robust in the presence of noise [24]. SVM can also be used with a variety of kernels. This study used linear, sigmoid, and RBF kernels, which transformed the low-dimensional input space into a higher-dimensional space, converting non separable problems into separable problems by adding more dimensions, which helps to build a more accurate classifier. Since the relationship between near misses and stress biomarker relative and absolute concentration is not very direct, increasing the dimensionality is likely what caused its high performance, particularly with the SVM with RBF kernels, which performed the best out of all models tested.

Interpreting LDA and SVM models can be difficult. It is hard to see the way the model makes its decisions by purely looking at the model weights and parameters. For this reason, although it has high accuracy, LDA and SVM models may not always be the right choice for clinical implementation. To mitigate this issue, we added an additional forest of trees for these models that allows for interpretation. This forest allows for feature importance ranking and shows the effect that each model input has on loss.

The Naïve Bayes method is a supervised learning algorithm based on Bayes theorem in machine learning [25]. Our NB model had a training accuracy of 79% and a test accuracy of 73% and although the accuracy was not the highest in comparison to the other models tested, its high computational efficiency [26], interpretability, and ability to predict in the presence of missing data [27] make it well suited to clinical implementation. Our NB model showed that the pre shift ratio of cortisol to DHEA was the most significant predictor of near misses in this dataset.

The KNN classifier is a non-parametric method that has the capacity to learn nonlinear relationships well and is clinically appealing, performing well in comparison to more complicated models [28]. The KNN model developed has a training accuracy of 76% and a validation accuracy of 73%. As it performed similarly to the NB model, it likely would not be the model of choice for this application, as NB models also afford significant computational efficiency along with other benefits not matched by the KNN. KNN models are additionally not as interpretable as NB models.

With this limited dataset, the SVM with RBF kernels had the best performance. Based on the discussions above, this is likely due to the ability of the SVM to find more indirect relationships by increasing the dimensionality of the data. However, considering all factors, alternative models, such as the NB, may be used instead, as SVM are black box algorithms that cannot be readily interpreted.

This study had several limitations. The algorithm was trained on a small dataset. which may mean overfitting and may not generalize well to other clinical settings. To improve on this in the future, a larger dataset should be used to train, with the train, test, and validation cohort not randomly split from a homogenous dataset but should be from separate clinics or of different caliber, to see the efficacy of the model in a more realistic setting. Additionally, the data collected did not have a control group to compare to.

This study found that cortisol, specifically pre-shift cortisol, was more important to the model than DHEA-S concentrations. The pre-shift cortisol to DHEA-S ratio was the most significant feature to model predictions. The feature importance rankings also found that the pre-shift cortisol to DHEA-S ratio was more significant than their post-shift counterpart. This suggests that interventions should be implemented that help emergency room physicians relax before they begin their shift, as this could reduce risk of errors and improve patient and physician outcomes.

The link between stress and cortisol is well researched, and cortisol is a reasonable biomarker of stress [29]. Cortisol has been linked to sleep loss [30] and is well shown to have neurotoxic effects after long term or repeated stress exposure [31]. Cortisol and anxiety have also been shown to have significant correlations to stress in sleep deprived medical residents [32].

By focusing on the ratio between these two hormones along with the absolute concentration of each, information about the preferential production of these hormones can be assessed. The cortisol to DHEA-S ratio has been used in previous studies and shown to increase with increased stress severity ratings [33]. Using the ratio also allowed us to determine the significance of the balance between cortisol and DHEA-S rather than merely focusing on each individual hormone. Past studies have also reported that separately, cortisol and DHEA have no significant correlation with certain physiological processes, such as sepsis, but together they had predictive power [34, 35], further validating this approach. The study suggests that future assessments of physician stress should focus on the cortisol to DHEA ratio along with absolute blood concentrations of these two biomarkers. Should these findings hold up in future larger confirmatory research, the cortisol to DHEA-S ratio pre-shift might be one measure to better define at-risk physicians prior to them initiating their often high-intensity and high-risk work in the emergency department.

## Conclusions

Once the model is further refined, biomarkers and clinical data might be used to predict risk of adverse events in clinical practices. The amount of cortisol and DHEA-S as well as the cortisol to DHEA-S ratio could be used proactively to assess risks for adverse events due to stress, with a focus on the cortisol to DHEA-S ratio. Future studies should use larger groups and relate these variables to administrative data in electronic medical records, such as treatment quality.

## Supporting information

S1 File Study dataset. This datafile includes the cortisol and DHEA-S blood concentrations and all data used for the study.
(XLSX)

## References

[1] Bakker AB, Costa PL. Chronic job burnout and daily functioning: A theoretical analysis. Burnout Research. 2014 Dec 1;1(3):112–9.

[2] Rotenstein LS, Torre M, Ramos MA, Rosales RC, Guille C, Sen S, et al. Prevalence of burnout among physicians: a systematic review. Jama. 2018 Sep 18;320(11):1131–50. doi: 10.1001/jama.2018.12777 30326495 PMC6233645

[3] West CP, Dyrbye LN, Shanafelt TD. Physician burnout: contributors, consequences and solutions. Journal of internal medicine. 2018 Jun;283(6):516–29. doi: 10.1111/joim.12752 29505159

[4] Stehman C, Testo Z, Gershaw R, Kellogg A. Burnout, Drop Out, Suicide: Physician Loss in Emergency Medicine, Part I. Western Journal of Emergency Medicine (Internet). 2019 Apr 23;20(3):485–94. 10.5811/westjem.2019.4.40970 PMC6526882 31123550

[5] Sprague J, Verona E, Kalkhoff W, Kilmer A. Moderators and mediators of the stress-aggression relationship: Executive function and state anger. Emotion. 2011;11(1):61–73. doi: 10.1037/a0021788 21401226

[6] Toker S, Melamed S. Stress, Recovery, Sleep, and Burnout. The Handbook of Stress and Health. 2017;168–85.

[7] Arnetz BB, Lewalski P, Arnetz J, Breejen K, Przyklenk K. Examining self-reported and biological stress and near misses among Emergency Medicine residents: a single-centre cross-sectional assessment in the USA. BMJ Open. 2017;7(8). doi: 10.1136/bmjopen-2017-016479 28814584 PMC5629729

[8] Toren O, Dokhi M, Ganz FD. Hospital nurses’ intention to report near misses, patient safety culture and professional seniority. International Journal for Quality in Health Care. 2021;33(1). doi: 10.1093/intqhc/mzab031 33620464

[9] Deo RC. Machine Learning in Medicine. Circulation. 2015 Nov 17;132(20):1920–30. doi: 10.1161/CIRCULATIONAHA.115.001593 26572668 PMC5831252

[10] Bzdok D, Altman N, Krzywinski M. Statistics versus machine learning. Nature Methods. 2018 Apr;15(4):233–4. doi: 10.1038/nmeth.4642 30100822 PMC6082636

[11] Kourou K, Exarchos TP, Exarchos KP, Karamouzis MV, Fotiadis DI. Machine learning applications in cancer prognosis and prediction. Computational and Structural Biotechnology Journal. 2015;13:8–17. doi: 10.1016/j.csbj.2014.11.005 25750696 PMC4348437

[12] Simes RJ. Treatment selection for cancer patients: application of statistical decision theory to the treatment of advanced ovarian cancer. Journal of chronic diseases. 1985 Jan 1;38(2):171–86. doi: 10.1016/0021-9681(85)90090-6 3882734

[13] Cruz JA, Wishart DS. Applications of machine learning in cancer prediction and prognosis. Cancer informatics. 2006 Jan;2:117693510600200030. PMC2675494 19458758

[14] Schafer KM, Kennedy G, Gallyer A, Resnik P. A direct comparison of theory-driven and machine learning prediction of suicide: A meta-analysis. PloS one. 2021 Apr 12;16(4):e0249833. doi: 10.1371/journal.pone.0249833 33844698 PMC8041204

[15] Bozorgmehr A, Thielmann A, Weltermann B. Chronic stress in practice assistants: An analytic approach comparing four machine learning classifiers with a standard logistic regression model. Plos one. 2021 May 4;16(5):e0250842. doi: 10.1371/journal.pone.0250842 33945572 PMC8096078

[16] Kaczor EE, Carreiro S, Stapp J, Chapman B, Indic P. Objective measurement of physician stress in the emergency department using a wearable sensor. In Proceedings of the Annual Hawaii International Conference on System Sciences. Annual Hawaii International Conference on System Sciences 2020 (Vol. 2020, p. 3729). NIH Public Access. PMC6996921 32015695

[17] Elliott ML, Belsky DW, Knodt AR, Ireland D, Melzer TR, Poulton R, et al. Brain-age in midlife is associated with accelerated biological aging and cognitive decline in a longitudinal birth cohort. Molecular psychiatry. 2019 Dec 10:1–0. 10.1038/s41380-019-0626-7 PMC7282987 31822815

[18] Zwain IH, Yen SSC. Dehydroepiandrosterone: Biosynthesis and Metabolism in the Brain. Endocrinology. 1999;140(2):880–7. doi: 10.1210/endo.140.2.6528 9927319

[19] Kroboth PD, Amico JA, Stone RA, Folan M, Frye RF, Kroboth FJ, et al. Influence of DHEA Administration on 24-Hour Cortisol Concentrations. Journal of Clinical Psychopharmacology. 2003;23(1):96–9. doi: 10.1097/00004714-200302000-00014 12544381

[20] Hastie T, Friedman J, Tisbshirani R. The Elements of statistical learning: data mining, inference, and prediction. Springer; 2017.

[21] Majd TM, Kalantari S, Shahraki HR, Nafar M, Almasi A, Samavat S, et al. Application of Sparse Linear Discriminant Analysis and Elastic Net for Diagnosis of IgA Nephropathy: Statistical and Biological Viewpoints. Iranian Biomedical Journal. 2018;22(6):374–84. doi: 10.29252/.22.6.374 29523019 PMC6305813

[22] Farago E, Chinchalkar S, Lizotte DJ, Trejos AL. Development of an EMG-Based Muscle Health Model for Elbow Trauma Patients. Sensors. 2019;19(15):3309. doi: 10.3390/s19153309 31357650 PMC6695912

[23] Shayan Z, Mezerji NMG, Shayan L, Naseri P. Prediction of Depression in Cancer Patients With Different Classification Criteria, Linear Discriminant Analysis versus Logistic Regression. Global Journal of Health Science. 2015;8(7):41. doi: 10.5539/gjhs.v8n7p41 26925900 PMC4965639

[24] Golpour P, Ghayour-Mobarhan M, Saki A, Esmaily H, Taghipour A, Tajfard M, et al. Comparison of Support Vector Machine, Naïve Bayes and Logistic Regression for Assessing the Necessity for Coronary Angiography. International Journal of Environmental Research and Public Health. 2020;17(18):6449. doi: 10.3390/ijerph17186449 32899733 PMC7558963

[25] Langarizadeh M, Moghbeli F. Applying Naive Bayesian Networks to Disease Prediction: a Systematic Review. Acta Informatica Medica. 2016;24(5):364. doi: 10.5455/aim.2016.24.364-369 28077895 PMC5203736

[26] Batista P, Pereira A. Biomarkers in Neurodegenerative Diseases: Cortisol. Journal of Molecular Biomarkers & Diagnosis. 2016;07(02).

[27] Heijden MVD, Velikova M, Lucas PJ. Learning Bayesian networks for clinical time series analysis. Journal of Biomedical Informatics. 2014;48:94–105. doi: 10.1016/j.jbi.2013.12.007 24361389

[28] Parry RM, Jones W, Stokes TH, Phan JH, Moffitt RA, Fang H, et al. k-Nearest neighbor models for microarray gene expression analysis and clinical outcome prediction. The Pharmacogenomics Journal. 2010;10(4):292–309. doi: 10.1038/tpj.2010.56 20676068 PMC2920072

[29] Çay M. The Effect of Cortisol Level Increasing Due to Stress in Healthy Young Individuals on Dynamic and Static Balance Scores. Northern Clinics of Istanbul. 2017. 10.14744/nci.2017.42103 PMC6371989 30859159

[30] Hirotsu C, Tufik S, Andersen ML. Interactions between sleep, stress, and metabolism: From physiological to pathological conditions. Sleep Science. 2015;8(3):143–52. doi: 10.1016/j.slsci.2015.09.002 26779321 PMC4688585

[31] Kamin HS, Kertes DA. Cortisol and DHEA in development and psychopathology. Hormones and Behavior. 2017;89:69–85. doi: 10.1016/j.yhbeh.2016.11.018 27979632

[32] Morales J, Yáñez A, Fernández-González L, Montesinos-Magraner L, Marco-Ahulló A, Solana-Tramunt M, et al. Stress and autonomic response to sleep deprivation in medical residents: A comparative cross-sectional study. Plos One. 2019;14(4). doi: 10.1371/journal.pone.0214858 30947295 PMC6448892

[33] Heaney JL, Carroll D, Phillips AC. Physical Activity, Life Events Stress, Cortisol, and DHEA: Preliminary Findings That Physical Activity May Buffer Against the Negative Effects of Stress. Journal of Aging and Physical Activity. 2014;22(4):465–73. doi: 10.1123/japa.2012-0082 24084142

[34] Arlt W, Hammer F, Sanning P, Butcher SK, Lord JM, Allolio B, et al. Dissociation of Serum Dehydroepiandrosterone and Dehydroepiandrosterone Sulfate in Septic Shock. The Journal of Clinical Endocrinology & Metabolism. 2006; 91(7):2548–54. doi: 10.1210/jc.2005-2258 16608898

[35] Butcher SK, Killampalli V, Lascelles D, Wang K, Alpar EK, Lord JM. Raised cortisol:DHEAS ratios in the elderly after injury: potential impact upon neutrophil function and immunity. Aging Cell. 2005;4(6):319–24. doi: 10.1111/j.1474-9726.2005.00178.x 16300484

